# Sorption of CO_2_, CH_4_ and Their Mixtures in Amorphous Poly(2,6-dimethyl-1,4-phenylene)oxide (PPO)

**DOI:** 10.3390/polym15051144

**Published:** 2023-02-24

**Authors:** Valerio Loianno, Antonio Baldanza, Giuseppe Scherillo, Pellegrino Musto, Giuseppe Mensitieri

**Affiliations:** 1 Department of Chemical, Materials and Production Engineering, University of Naples Federico II, Piazzale Tecchio 80, 80125 Naples, Italy; 2 Institute for Polymers, Composites and Biomaterials, National Research Council of Italy, Via Campi Flegrei 34, 80078 Pozzuoli, Italy; 3 Reference Centre for Transformation Technology of Polymeric and Composite Materials, Italian Interuniversity Consortium on Materials Science and Technology (INSTM), Piazzale Tecchio 80, 80125 Naples, Italy

**Keywords:** mixed gas sorption, glassy polymers, FTIR spectroscopy, thermodynamic modelling, NRHB

## Abstract

Sorption of pure CO_2_ and CH_4_ and CO_2_/CH_4_ binary gas mixtures in amorphous glassy Poly(2,6-dimethyl-1,4-phenylene) oxide (PPO) at 35 °C up to 1000 Torr was investigated. Sorption experiments were carried out using an approach that combines barometry with FTIR spectroscopy in the transmission mode to quantify the sorption of pure and mixed gases in polymers. The pressure range was chosen to prevent any variation of the glassy polymer density. The solubility within the polymer of the CO_2_ present in the gaseous binary mixtures was practically coincident with the solubility of pure gaseous CO_2_, up to a total pressure of the gaseous mixtures equal to 1000 Torr and for CO_2_ mole fractions of ~0.5 mol mol^−1^ and ~0.3 mol mol^−1^. The Non-Equilibrium Thermodynamics for Glassy Polymers (NET-GP) modelling approach has been applied to the Non-Random Hydrogen Bonding (NRHB) lattice fluid model to fit the solubility data of pure gases. We have assumed here that no specific interactions were occurring between the matrix and the absorbed gas. The same thermodynamic approach has been then used to predict the solubility of CO_2_/CH_4_ mixed gases in PPO, resulting in a deviation lower than 9.5% from the experimental results for CO_2_ solubility.

## 1. Introduction

The use of combustible gases has been increasing in the last few decades and this trend is expected to persist in the near future. Indeed, in 2021, the inland demand of natural gas (NG) in the EU and the US increased by 4.3% and 0.5%, respectively, compared to those in 2020 [1,2]. Glassy polymeric membranes already play an important role in the purification of methane from NG and, usually, cellulose acetate or polyimide membranes are used to remove carbon dioxide [3]. Their CO_2_/CH_4_ selectivity is high when measured by pure gas permeation tests but is severely depleted when field conditions are considered, even by 80% [4]. This behavior is attributed to enhanced ageing phenomena when hydrocarbon or water traces are present in the feed line and to competitive sorption phenomena or induced swelling produced in the case of mixed gases [5]. Conversely, poly(2,6-dimethyl-1,4-phenylene) oxide (PPO) has shown satisfactory separation performances for CO_2_/CH_4_ mixtures and a great thermal stability, having a glass transition temperature (*T_g_*) of approximately 210 °C. Chenar et al. [6] also demonstrated a good chemical stability of this polymer in the presence of water. In fact, they conducted permeation measurements on hollow fiber modules made of PPO by fixing the pressure of the feed at 7.9 bar and the temperature at 30 °C. The authors conducted dry and wet experiments; in the former, the feed gas mixture only consisted of pure methane or a mixture of carbon dioxide and methane, while in the latter, the same streams were saturated with water vapor. They found a small reduction in the selectivity from 9.0 (dry regime) to 8.0 (wet regime) at a feed CO_2_ concentration of 5% by volume. When the concentration of CO_2_ reached 25% by volume, the water content produced no effect on the selectivity, being equal to ~8.5 in both regimes.

Previously, Story and Koros measured the solubility of mixed CO_2_/CH_4_ in PPO [7]. They observed that the solubility of each gas was reduced in the polymer with respect to the measurements for the pure gas sorption experiment conducted at pressure values equal to the values of the partial pressure of the gas in the mixture. Similar to this, we studied the sorption of pure CO_2_ and CH_4_ and their mixtures in PPO at 35 °C up to 1000 Torr and present both experimental results, gathered by combining barometric and spectroscopic approaches, and their interpretation based on a theory rooted in statistical thermodynamics.

The closed volumetric method is commonly used to measure the solubility of mixed gases in polymers [7,8,9]. The measurement of the gas phase composition at sorption equilibrium is conducted ex situ with gas chromatography (GC); the volumes must be precisely calibrated and an accurate Equation of State is required to evaluate the number of moles of each gas species [10]. Recently, we introduced a new experimental technique coupling barometry and FTIR Spectroscopy in the transmission mode to measure the solubility of binary gas mixtures in rubbery polymers [11]. Specifically, we studied the sorption of mixed CO_2_/CH_4_ in Polydimethylsiloxane (PDMS) at ambient temperatures and were able to evaluate the solubility of CO_2_ in PDMS up to 9 bar of total pressure and CO_2_ mole fraction of ~0.5 mol mol^−1^. With this new technique, the classical closed volumetric method can be used by monitoring the concentration depletion of each gas species in situ with the corresponding IR signal. Alternatively, the solubility of each absorbed species can be evaluated directly in the polymer phase by isolating their own IR signals with difference spectroscopy. Hong et al. previously used this second approach with FTIR Spectroscopy in the ATR mode [12]. They studied the sorption of a binary vapor mixture of Methyl Ethyl Ketone and Toluene in Polyisobutylene, and since the gas phase composition could not be measured, it was fixed during the sorption test. The approach we have introduced has the important advantage of being relatively simple to perform, thus avoiding the complexity of the experimental techniques currently used to measure the solubility of mixed gases in glassy polymers that hampers the evaluation of the performances and the selection of the best performing material for a specific gas separation application [13].

Regarding the theoretical approaches capable of predicting accurately the transport properties of glassy polymers in contact with multicomponent gas mixture, as pointed out by Minelli and Sarti, they must be still fully developed, and a few good results have been obtained so far by applying the Non-Equilibrium Thermodynamics for Glassy Polymers (*NET-GP*) to extend the use of compressible lattice fluid (*LF*) models, originally developed to describe the behavior of rubbery polymers and to deal with the case of glassy polymers [4,5,6,7,8,9,10,11,12,13,14,15,16]. Minelli et al. followed this approach by extending the Sanchez and Lacombe *LF* (*SL*) model to predict the sorption uptake at thermodynamic equilibrium of CO_2_/CH_4_ in PPO and CO_2_/C_2_H_4_ and CO_2_/N_2_O in Poly(methyl methacrylate) (PMMA), after retrieving each polymer–penetrant mean field interaction parameter from the fitting pure gas solubility data. Later, we used the same approach to extend the Non-Random Hydrogen Bonding (*NRHB*) lattice fluid model proposed by Panayiotou et al. [17,18]. This model overcomes some important limitations of the *SL* theory [19,20,21,22]. In fact, the *NRHB* model, different from the *SL* approach, is thermodynamically consistent if applied to multicomponent fluid mixtures, providing chemical potential expressions that converge, in the ideal gas limit, to the corresponding expressions for ideal gas mixtures [23]. Consequently, *NETGP-NRHB* model does not suffer the inconsistency of the multicomponent external fluid phase, which is naturally inherited by the *NETGP-SL* model [16]. Moreover, the *NRHB* model and its *NETGP* extension account for the non-randomicity of contacts between the polymer repeat units, the penetrant molecules and the empty sites as well as for possible specific self- and cross-interactions in the penetrant–polymer mixture (e.g., self and cross hydrogen bonding or Lewis acid –base interactions) [19]. In the present context, in view of the characteristics of the system under scrutiny, we have not accounted for the presence of specific interactions considering only the non-randomicity of contacts. In the following, the modelling approach, obtained by applying the *NET-GP* procedure to the *NRHB* model deprived of the *HB* contribution, will be referred to as the *NETGP-NR* theory. 

To evaluate the efficacy of the *NET-GP* extension applied to a *LF* theoretical framework for predicting mixed gas sorption in a polymer, a broad spectrum of thermodynamic conditions and polymer–penetrant systems should be investigated [15]. Indeed, for this aim, the availability in the literature of mixed gas solubility data in polymers is still inadequate.

Moving from these considerations, our goals are multiple. First, we extend the experimental technique coupling Barometry and FTIR Spectroscopy in the transmission mode, applied till now only to the case of rubbery polymers, to the case of glassy polymers by investigating the sorption of pure CO_2_, CH_4_ and their mixtures in PPO. Second, we interpret the solubility data of pure CO_2_ and CH_4_ in PPO with the *NETGP-NR* theory. Last, we use the same model to predict the mixed CO_2_/CH_4_ solubility in PPO and compare these results with the experimental data on mixture sorption.

## 2. Theoretical Background

### 2.1. The NETGP-NR Model

*NETGP-NR* model was developed by our group [16,19] with the aim of modelling the equilibrium, at fixed *P* and *T*, between an amorphous polymer–penetrant mixture “frozen” in a glassy state and a fluid consisting of a mixture of low molecular weight penetrants. The polymer is assumed to be not soluble in the fluid phase. This should be considered as a pseudo-equilibrium in view of the non-equilibrium state of the glassy polymer mixture that is at a temperature far below its glass-to-rubber transition temperature, *T_g_*. In such a condition, the mass density of the polymer is kinetically locked at an out-of-equilibrium value. If the low molecular weight penetrants are light gases at low pressure, as is the case in the present investigation, the polymer density can be identified with that of the unpenetrated polymer, $\rho_{p,0}$, right before the start of the sorption process. This value is a function of the previous thermomechanical history of the unpenetrated polymer sample and, being an out-of-equilibrium value, its value cannot be provided by any equation of state (EoS). It is commonly retrieved from experiments and it represents a key parameter of the model. In short, the phase equilibrium is described by using the expressions for the chemical potentials of the components of the glassy polymer–penetrants mixture provided by the *NETGP-NR* model while the expressions for the chemical potentials of the components of the fluid phase are simply provided by the corresponding equilibrium thermodynamics model, i.e., the Non-Random lattice fluid model.

Consistently with the II law of thermodynamics, the described phase Pseudo-Equilibrium conditions are still dictated by the equivalence of the chemical potentials, $\mu_{i}$, of each component *i* present in both the coexisting phases [19,24,25] (the meanings of the symbols of Equation (1) and of all the following equations are reported in the ‘List of symbols’ section at the end of the manuscript):

(1) $$\mu_{i,pol}^{NE}\left(T,\;x_{1}^{NE},\ldots ,x_{m-1}^{NE},\rho_{p,0}\;\right)=\mu_{i,\;\;ext}^{EQ}\left(T,P,x_{1,ext},\ldots ,x_{m-2,ext}\right)\;\mathrm{for}\;i=1,\ldots m-1$$

The equivalence of chemical potentials is only imposed for the penetrants since no polymer is assumed to be present within the external fluid phase. In Equation (1), subscript *pol* and *ext* stand for polymer–penetrant and external penetrant phase, respectively. Moreover, *T* and *P* represent the uniform temperature and pressure fields of the multicomponent biphasic system, respectively; *x_i_* stands for the molar fraction of penetrant species *i*, *m* − 1 represents the total number of types of penetrants considered in the system, $x_{i}^{NE}$ represents the composition of each component within the polymer phase at the Pseudo-Equilibrium of phase condition and the superscripts *NE* and *EQ* refer to non-equilibrium and equilibrium conditions, respectively.

We recall here the dimensionless expression of the chemical potential of the *i*-th penetrant provided by the *NETGP-NR* model [16]:

(2) $$\frac{\mu_{i,pol}^{NE}}{RT}=ln\frac{\Phi_{i}}{\delta_{i}\;r_{i}}+ln\tilde{\rho }-r_{i}ln\left(1-\tilde{\rho }\right)-\frac{z}{2}r_{i}\left(\frac{q_{i}}{r_{i}}-1\right)ln\left(1-\tilde{\rho }+\frac{q}{r}\tilde{\rho }\right)+\frac{z\;q_{i}}{2}\left[ln\Gamma_{ii}-\frac{r_{i}}{q_{i}}ln\Gamma_{00}\right]-\frac{q_{i}}{{\tilde{T}}_{i}}$$

(3) $$\frac{\Gamma_{ij}{}^{2}}{\Gamma_{ii}\;\Gamma_{jj}}=exp\left(-\frac{\Delta \varepsilon_{ij}}{RT}\right)\;\;\;\;\;\;\;\;for\;each\;i,j=0,\;1,\ldots ,\;m\;and\;j>i$$

(4) $$\overset{m}{\underset{j=0}{\sum }}\Theta_{j}\Gamma_{ij}=1\;\;\;\;\;\;\;\;\;\;\;\;\;\;\;\;\;\;\;for\;each\;i=0,\;1,\ldots ,\;m$$

The following set of dimensionless equations provides instead the equilibrium chemical potential of the *i*-th penetrant:

(5) $$\frac{\mu_{i}^{eq}}{RT}=ln\frac{\Phi_{i}}{\delta_{i}\;r_{i}}-r_{i}\overset{m}{\underset{j=1}{\sum }}\frac{\Phi_{j}l_{j}}{r_{j}}+ln\tilde{\rho }+r_{i}\left(\tilde{v}-1\right)ln\left(1-\tilde{\rho }\right)\;-\frac{z}{2}r_{i}\left(\tilde{v}-1+\frac{q_{i}}{r_{i}}\right)ln\left(1-\tilde{\rho }+\frac{q}{r}\tilde{\rho }\right)\;\;\;\;\;\;\;\;\;\;\;\;\;\;\;\;\;\;+\frac{z\;q_{i}}{2}\left[ln\Gamma_{ii}+\frac{r_{i}}{q_{i}}\left(\tilde{v}-1\right)ln\Gamma_{00}\right]+r_{i}\frac{\tilde{p}\;\tilde{v}}{\tilde{T}}-\frac{q_{i}}{{\tilde{T}}_{i}}$$

(6) $$\frac{\Gamma_{ij}{}^{2}}{\Gamma_{ii}\;\Gamma_{jj}}=exp\left(-\frac{\Delta \varepsilon_{ij}}{RT}\right)\;\;\;\;\;\;\;\;for\;each\;i,j=0,\;1,\ldots ,\;m\;-1\;and\;j>i$$

(7) $$\sum_{j=0}^{m}\Theta_{j}\Gamma_{ij}=1\;\;\;\;\;\;\;\;for\;each\;i,j=0,\;1,\ldots ,\;m\;-1$$

(8) $$\tilde{P}+\tilde{T}\left[ln\left(1-\tilde{\rho }\right)-\tilde{\rho }\left(\sum_{i=1}^{m}\Phi_{\dot{i}}\frac{l_{\dot{i}}}{r_{\dot{i}}}\right)-\frac{z}{2}ln\left(1-\tilde{\rho }+\frac{q}{r}\tilde{\rho }\right)+\frac{z}{2}ln\Gamma_{00}\right]=0$$

$\Gamma_{ij}$ represents the non-random factor, and Equations (3), (4), (6) and (7) are the minimization and balance of contact expressions for the polymer–penetrant non-equilibrium phase (Equations (3) and (4)) and the external multicomponent penetrant equilibrium phase (Equations (6) and (7)). Equation (8) represents the EoS of the model, which holds only in the external penetrant phase and provides the equilibrium the reduced density of the mixture, $\tilde{\rho }$, to be adopted in the set of Equations (5)–(7). 

The equations for a pure component phase (in this case, *m = i = 1* holds) are consistently obtained by setting $\Phi_{1}=1$, where $\Phi_{i}$ represents the fraction of mers of species *i*. The dimensionless form of the *NETGP-NR* model’s Equations (4)–(10) are obtained by properly scaling the temperature, pressure and phase density variables in each phase by using the related lattice fluid parameters [17,18,19].

Each component is characterized by four composition-independent lattice fluid parameters that are commonly estimated by non-linear regressions of equilibrium thermophysical properties of the pure components. Vapor–liquid equilibrium data are in general used in the case of low molecular weight compounds while equilibrium dilatometric data are adopted in the case of polymers. The first two are an “enthalpic contribution” parameter, $\varepsilon_{i,h}^{*}$, and an “entropic contribution” parameter, $\varepsilon_{i,s}^{*}$, that are combined to calculate the “mean field interaction energy” per molar segment, $\varepsilon_{i}^{*}$. A third parameter, $v_{i,sp,0}^{*}$, represents the temperature-independent contribution to the closely packed specific volume of the pure component *i*, $v_{i,sp}^{*}$. The values of the parameters $\varepsilon_{i,h}^{*}$, $\varepsilon_{i,s}^{*}$ and $\;v_{i,sp,0}^{*}$ are retrieved, for each component *i*, by fitting its equilibrium thermophysical properties. Finally, the fourth composition independent *LF* parameter, associated with component *i*, is represented by the *shape factor*, *s_i_*, defined as the ratio of molar surface to molar volume, *s_i_ = q_i_/r_i_*. To reduce the number of optimization parameters involved in the fitting procedure of equilibrium, thermophysical data *s_i_* is commonly estimated through the *UNIFAC* group contribution method, and this is also the approach followed in the present investigation [26].

Once the four *LF* parameters have been determined for each pure component, the scaling parameters for a mixture of these components are univocally evaluated according to the mixing rules of the adopted *LF* model. These scaling parameters are a function of concentration and of the pure component *LF* parameters [17,18,19]. 

Regarding the *LF* scaling energy, the following mixing rule is assumed (each $\theta_{i}$ is function of concentration) [17,18,19]:

(9) $$\varepsilon^{*}=\overset{m}{\underset{i=1}{\sum }}\overset{m}{\underset{j=1}{\sum }}\theta_{i}\theta_{j}\sqrt{\varepsilon_{i}^{*}\varepsilon_{j}^{*}}\;\left(1-k_{ij}\right)$$

Equation (9) introduces an additional dimensionless parameter, $k_{ij}$, associated with each couple of components (*i*,*j*) involved in the multicomponent mixture. It measures the departure from the geometric mean rule for the corresponding *LF* (“mean field”) interactional energy. $k_{ij}$ is commonly assumed to be a pure binary parameter, only a function of the nature of binary interactions of the couple of components *i - j*. Consequently, it can be obtained by a non-linear regression of equilibrium and/or Pseudo-Equilibrium of phase properties of the corresponding binary system. This approach has been followed in the present investigation: the value of $k_{ij}$ for the penetrant binary phase of interest has been retrieved by *VLE* data of the methane/carbon dioxide system while the values of $k_{ij}$ for the couples PPO-methane and PPO-carbon dioxide are respectively evaluated by non-linear regressions of the corresponding Pseudo-of Equilibrium solubility data. 

The reduced density of the out of equilibrium glassy mixture $\;(\tilde{\rho }$), to be used in Equations (2)–(4), cannot be obtained from the NR equation of state but it is dictated by the out of equilibrium value of $\rho_{p,0}$:

(10) $$\tilde{\rho }=\rho_{p,0}/\left(\omega_{p}\rho^{*}\right)$$

where $\omega_{p}$ is the mass fraction of polymer and $\rho^{*}$ is the closed-packed density of the polymer–penetrant mixture, which is dictated by the corresponding mixing rules of the *LF* model for the numbers of occupied mers for component *i* [17,18,19].

### 2.2. Solution Diffusion Model of Small Molecules in Polymers

The transport of low molecular mass species, such as gases or vapors, in dense polymeric membranes is commonly described as a two-step process involving the dissolution of the guest within the host matrix and the subsequent diffusion through it [27]. In a pure gas permeation test through a plane polymeric sheet, if the gas pressure and concentration of gas molecules solubilized within the polymer (*P*, *C*) at the upstream side of the membrane are far greater than at the downstream side, the steady state mean permeability ($\bar{Perm}$) of the gas in the material may be expressed as [28]:

(11) $$\bar{Perm}=\bar{D}\times S$$

if one assumes that the constitutive equation for mass transport is provided by the Fick’s law [28]. Here, *S* is the apparent solubility coefficient and $\bar{D}$ is the effective diffusivity coefficient. *S* is defined as the ratio of the gas solubility in the polymer corresponding to the upstream pressure of the system. The mutual diffusion coefficient *D* may be concentration dependent and $\bar{D}$ is an estimate of the average diffusivity in the concentration range [$C^{d}$,$C^{up}$], where $C^{d}$ and $C^{up}$ represent, respectively, the concentration within the polymer in contact with the gas at the downstream and upstream side of the membrane, calculated as follows:

(12) $$\bar{D}=\frac{1}{C^{up}-C^{d}\;}\int_{C^{d}\;}^{C^{up}}D\left(x\right)\;dx$$

*S* and *D* take into account the host/guest affinity and the guest mobility within the host matrix, respectively. The polymer capability of separating two low molecular weight components of a gas mixture (identified here with the subscript ‘*i*’ and ‘*j*’) is usually evaluated from the ideal selectivity ($\alpha^{id}$) equal to:

(13) $$\alpha^{id}=\frac{Perm_{i}}{Perm_{j}}=\frac{{\bar{D}}_{i}S_{i}}{{\bar{D}}_{j}S_{j}}=\alpha_{S}^{id}\cdot \alpha_{D}^{id}$$

where $\alpha_{S}^{id}$ and $\alpha_{D}^{id}$ are, respectively, the solubility and diffusivity of ideal selectivity, provided the diffusivity is invariant in the range [$C^{d}$,$C^{\mathrm{up}}$]. However, when designing a gas mixture separation apparatus based on permeation through a polymer membrane, the real selectivity should be considered. To this aim, we still consider the case where the upstream partial pressure of each component is significantly higher than the corresponding downstream ones. The solubility coefficient within the polymer phase of the gaseous species *i* is estimated at the corresponding upstream pressure ($P_{i}^{up}$), i.e.,:

(14) $$S_{i}^{mix}=C_{i}^{up}/P_{i}^{up}$$

and, therefore, the solubility selectivity is expressed as:

(15) $$\alpha^{mix}=S_{i}^{mix}/S_{j}^{mix}$$

Conversely, the diffusion process occurring in a sorption experiment of a penetrant in a polymer plane sheet (i.e., when symmetrical boundary conditions are imposed) can be described in terms of evolution with time of the total mass of absorbed penetrant. This can be calculated by integrating the one-dimensional differential mass balance over the thickness of the polymer film. Symmetrical boundary conditions are imposed: the concentration of the penetrant within the polymer at both surfaces in contact with the external phase at time = 0 are fixed at the value $C_{i}^{\infty }$, that is the value dictated by the sorption equilibrium. The initial condition is that the concentration of penetrant is uniform within the polymer, $C_{i}^{0}$ (this value is equal to zero if the penetrant is initially not present within the polymer sample). If the constitutive expression for mass flux is Fickian, with a diffusivity independent from the penetrant concentration, the evolution with time of the mass of the absorbed penetrant (*M*(*t*)) in a plane sheet of thickness *L*, as obtained by solving the differential mass balance, can be expressed as [28]:

(16) $$\frac{M\left(t\right)}{M_{\infty }}=1-\overset{\infty }{\underset{n=0}{\sum }}\frac{8}{{\left(2n+1\right)}^{2}\pi^{2}}exp\left(-\frac{D\left(2n+1\right)\pi^{2}t}{L^{2}}\right)$$

where $M_{\infty }$ is the mass of penetrant absorbed when the sorption equilibrium with the external phase has been asymptotically attained. In Equation (16), it is assumed that the specimen thickness *L* and the boundary conditions are invariant during sorption.

In the following, ${\bar{C}}_{i}$ indicates the arithmetic average concentration during a sorption experiment (i.e., determined as ($C_{i}^{\infty }+C_{i}^{0})/2$) and $\bar{D}$ in Equation (16) is intended as calculated at $C={\bar{C}}_{i}$. 

## 3. Materials and Methods

### 3.1. Materials

**Figure 1:**
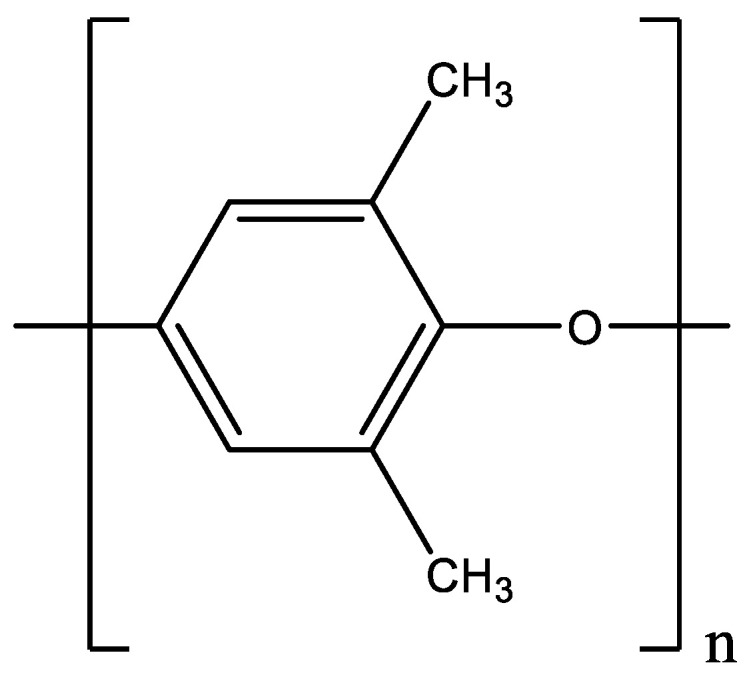
Repeating unit of Poly(2,6-dimethyl-1,4-phenylene)oxide.

Poly(2,6-dimethyl-1,4-phenylene)oxide (PPO, repeating unit reported in Figure 1) Ultra High P6130 grade (Mw = 350,000 g mol^−1^) was purchased from Sabic (Riyadh, Saudi Arabia). Sigma-Aldrich (Milan, Italy) supplied chloroform (CHCl_3_, purity ≥ 99.9%) and acetonitrile (ACN). Amorphous PPO films were prepared by solution casting from a 0.5%wt CHCl_3_ solution at T = 60 °C. The absorbed CHCl_3_ was removed from the cast films by ACN guest sorption/desorption at room temperature. The density of amorphous PPO films was measured by flotation in a CaCl_3_ aqueous solution and is equal to 1.063 g cm^−3^. Sol Spa (Monza, Italy) supplied carbon dioxide with a molar fraction purity of 999,950 μmol mol^−1^. Nippon Gases Industrial Sud S.r.l. (Naples, Italy) supplied methane with a molar fraction purity of 999,995 μmol mol^−1^.

### 3.2. Methods

#### 3.2.1. Closed Volume–Variable Pressure Apparatus

**Figure 2:**
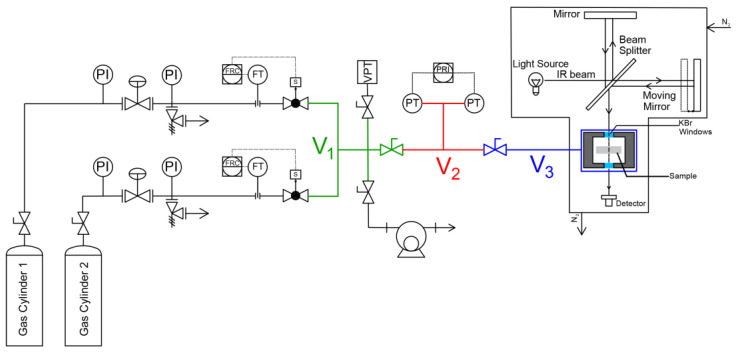
Schematic diagram of the apparatus used to conduct pure and mixed gas sorption experiments. The symbols V_1_, V_2_ and V_3_ identify the three closed volumes of the apparatus.

Integral and differential sorption experiments of pure CO_2_, CH_4_ and their mixtures in PPO were conducted in a closed volume variable pressure system similar to the one described by Loianno et al. [11]. The apparatus is schematically represented in Figure 2. It consists of three chambers indicated with the symbols V_1_, V_2_ and V_3_, whose volumes were calibrated with the Burnett expansion method and were equal to 15.87 cm^3^, 35.21 cm^3^ and 61.13 cm^3^, respectively. The latter was 44.71 cm^3^ by means of stainless-steel spheres. The three chambers were separated by shut-off valves type 4H-V-51 from Swagelok (Nordival S.r.l., Bs, Italy). The connections between service lines were realized using VCR fittings from Swagelok to ensure that the system was leak proof. Chamber 2 was equipped with two Baratron 121A pressure transducers from MKS Instruments (Andover, MA, USA), respectively, with a full-scale pressure range of 100 Torr and 1000 Torr. Their resolution was equal to 0.01 Torr and 0.1 Torr, respectively, and both had an accuracy of 0.5% of the reading. To guarantee temperature control, chamber 3 was jacketed and circulating water was supplied by a HAAKE F6 thermal bath (HAAKE Thermo Fisher Scientific, Breda, Belgium). Chambers 1 and 2 were kept at ambient temperature that was measured with a HD9215 thermometer from RS (Milan, Italy) equipped with a PT100 temperature transducer (resolution 0.1 °C, uncertainty ±0.2 °C). Pure gas sorption tests were conducted by excluding chamber 1. Mixed gas sorption tests were instead conducted by including chamber 1. In this case, the gas mixture was prepared using mass flow controller type GM50A-013102RMM020 from MKS Instruments (full scale volumetric flow range of N_2_: 100 ${\mathrm{cm}}_{\mathrm{STP}}^{3}$), following the protocol proposed by Loianno and Mensitieri [29].

To perform a static sorption test, the gas mixture of desired composition was first prepared in chambers 1 and 2 and was subsequently let to expand in chamber 3. Conversely, to perform a dynamic sorption test, the two gases, at the desired molar ratio, were introduced at the same time in the whole system, still using the mass flow controllers, and sorption occurred while the system was being filled to reach the desired pressure.

#### 3.2.2. FTIR-Spectroscopy in the Transmission Mode

During the sorption test, both the pressure signal and the IR spectrum were collected simultaneously. The IR spectroscopy measurement was performed in transmission mode in chamber 3. Two flat and coplanar KBr windows (4 mm thickness each) were aligned with the sample and allowed the IR beam to pass through the chamber. The chamber was made leak proof by placing Viton O-rings between the IR windows and their seats. The sample was cut into multiple pieces of the same thickness (63 μm), one of which was placed along the optical path of the IR beam. A spectrometer Spectrum 100 from Perkin Elmer (Norwalk, CT, USA) was used to collect the IR spectrum. It was equipped with a wide band deuterated triglycine sulfate detector working at room temperature and an interferometer including a germanium/KBr beam splitter. The detector had a wavelength response ranging from the near to the far infrared. The IR spectra collected at sorption thermodynamic equilibrium were averages of 32 coadded scans at 2 and 4 cm^−1^ and with a scan frequency of 5 s per spectrum. During detection of sorption kinetics, IR spectra were collected at 4 cm^−1^ with a scan frequency of 1 s per spectrum.

#### 3.2.3. Performing a Sorption Experiment

In a typical pure gas sorption test, chamber 2 was filled up to a specific pressure at ambient temperature and the gas was then expanded into chamber 3 whose temperature was kept at 35.00 ± 0.02 °C. Chamber 3 was initially either under high vacuum (integral test) or filled with gas at thermodynamic equilibrium with the polymer phase prior to the gas expansion (differential test). The solubility of the gas absorbed within the polymer was retrieved from a mole balance over the gas phase by evaluating the gas concentration depletion through barometry. It was assumed that the pressure is uniform throughout the apparatus and that the temperature displayed a step change at the border between chambers 2 and 3. The concentration of the gas was evaluated from the NIST Standard Reference Database 69: NIST Chemistry WebBook [30]. Multiple pieces of PPO were inserted into the volume of the measuring chamber to reduce the uncertainty of the solubility measurement. The total mass of PPO sample introduced into the measuring chamber was 0.3491 g.

In the case of mixture sorption, static sorption tests were performed up to pressures equal to around 0.7 atm. At higher pressures, dynamic sorption tests were performed. The amount of sorbed gas was evaluated via IR transmission spectroscopy. In particular, the concentration of carbon dioxide absorbed within the amorphous PPO was estimated using the IR signal at 3692 cm^−1^ associated with absorbed CO_2_ (see Section 4.1). Calibration of this signal was performed using absorbance vs. concentration data collected for the case of sorption of pure CO_2_ (see data and related discussion reported in Section 4.1). Unfortunately, since no signals could be used to quantify the concentration of CH_4_ sorbed at equilibrium, only data for the concentration of absorbed CO_2_ were reported in the case of experiments performed on CO_2_/CH_4_ mixtures. 

## 4. Results and Discussion

### 4.1. Sorption of Pure CO_2_ and CH_4_ in PPO

**Figure 3:**
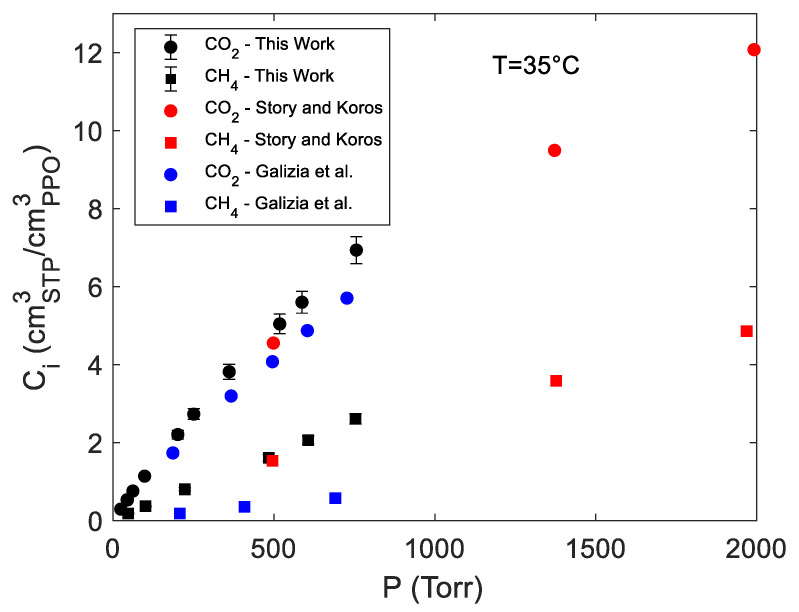
Sorption isotherms of pure CO_2_ and CH_4_ in amorphous PPO from the present investigation compared with the literature data [7,31].

Sorption isotherms at 35 °C of pure carbon dioxide and methane in amorphous PPO are reported in Figure 3 in terms of gas concentration within the polymer vs. gas pressure. In the same figure, experimental data obtained in this study are compared with analogous data available in the literature [7,31]. An excellent agreement was found with the data from Story and Koros, as was expected in view of the same sample preparation protocol adopted and the close values of the density of the samples (respectively, 1.068 g cm^−3^ and 1.063 g cm^−3^ for our samples and for the samples used by Story and Koros) [7,32]. 

Conversely, the solubility data reported by Galizia et al. [31] are systematically lower than the ones we obtained. In particular, in the case of carbon dioxide and methane average reductions of the solubility coefficients of 20% and 76% were observed, respectively. This is likely due to the different method of preparation of the samples that, in [31], were melted at 290 °C and then compression molded, thus, resulting in a film density equal to 1.016 g cm^−3^, significantly lower than that of the sample used in the present investigation.

**Figure 4:**
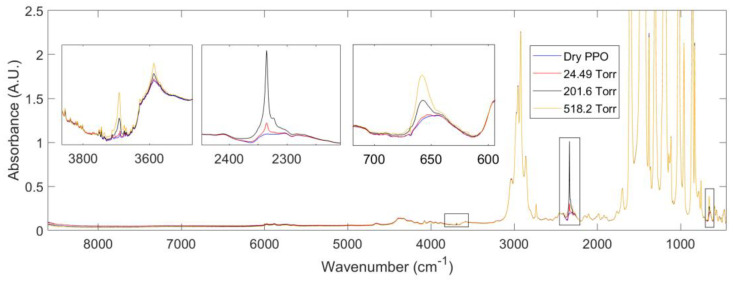
Absorbance spectra at 35 °C collected at sorption equilibrium after removal of the gas phase spectrum at 2 cm^−1^ resolution. The insets show the frequency regions in which carbon dioxide absorbs IR light within the polymer phase. In the range [2200,2450] cm^−1^, the spectrum at 518.2 Torr is saturated and is not reported for the sake of clarity.

During sorption, IR spectra of the polymer sample with sorbed gas molecules were also acquired. We first discuss the signals collected at sorption equilibrium for the amorphous PPO–CO_2_ system. In Figure 4, the absorbance spectrum of neat polymer and those of the PPO–CO_2_ system equilibrated at different pressures of CO_2_ are reported. Difference Spectroscopy was used to remove the contribution of the gas phase from the overall spectrum, so that the resulting spectra only consisted of the signals related to the polymer and the CO2 absorbed within it. Three frequency regions were identified where carbon dioxide absorbs IR light: the bending vibration at 659 cm^−1^; the antisymmetric stretching vibration at 2336 cm^−1^ and two overtones at 3586 and 3692 cm^−1^. The peak at 659 cm^−1^ showed the strongest absorptivity and was saturated above 250 Torr. In the range [250,1000] Torr, the overtone at 3692 cm^−1^ was the most suitable signal for a quantitative analysis. Indeed, its absorptivity was the greatest among the two identified overtones. Further, the absorbance uncertainty was lower in the range [3400,3900] cm^−1^ than in [600,700] cm^−1^ due to the greater transparency of the KBr windows of the measuring cell.

**Figure 5:**
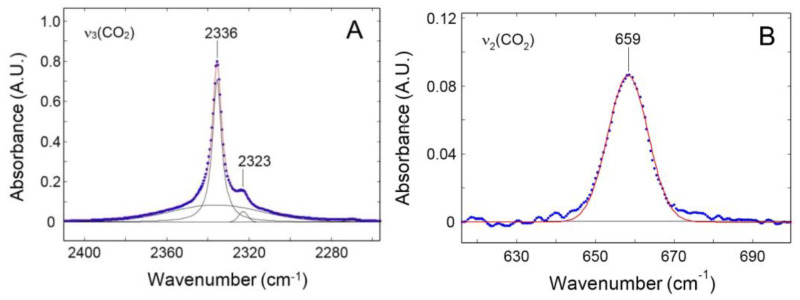
Band shape analysis of the ν_3_ (**A**) and ν_2_ (**B**) modes of CO_2_ sorbed in PPO. The spectrum, obtained by Difference Spectroscopy, refers to the test performed at 201.6 Torr. Blue dots: experimental data. Red line: simulated profile. Black lines: resolved components.

Difference Spectroscopy was again used to obtain the spectra of sorbed CO_2_. The band shape analysis of the signals produced by sorbed CO_2_ provides information on the interactions of the probe molecule with the surrounding environment (see Figure 5).

The ν_3_ profile represented in Figure 5A displayed a secondary maximum at 2323 cm^−1^. This feature was due to a non-fundamental transition [a (ν_3_ + ν_2_)–ν_2_ hot-band in Fermi resonance with the neighboring peak] and was to be neglected after proper resolution [33]. The main band, centered at 2336 cm^−1^, had a complex shape which, according to the theory of vibrational relaxation, can be simulated with high accuracy (R^2^ = 0.9988) by the sum of a Gaussian and a Lorentzian function, both centered at the peak maximum [34,35]. The composite band shape was originated by the probe dynamics within the molecular environment, in particular, free rotation in the early stages of the relaxation process (0.2−1.0 ps), which produces the Gaussian component, and random rotational diffusion at later stages (the so-called Debye regime), from which the Lorentz component arises. The pronounced Gaussian component (44.6% of the total band area) demonstrated a significant free-rotation regime, which, in turn, suggests that the probe is interrogating an essentially inert environment. 

The CO_2_ bending mode (ν_2_) is equally informative: this transition is degenerate for an isolated molecule but when an interaction takes place, a distortion of the linear configuration is produced which removes the original $D_{\infty h}$ symmetry. This effect activates distinct in-plane and out-of-plane modes and the signal splits in two fully or partially resolved components, depending on the degree of distortion. The occurrence of a two-component band shape provides a clear signature of an existing interaction between the probe and active sites on the polymer backbone and has been detected in numerous matrices including PMMA, poly(butyl methacrylate), poly(vinyl acetate), poly(vinyl fluoride) and, more recently, in an amorphous polyetherimide [33]. In the present case, the ν_2_ ban shape was symmetrical and can be reliably simulated by a single Gaussian function (Figure 5B), which confirms the absence of vibrationally detectable interactions. 

**Figure 6:**
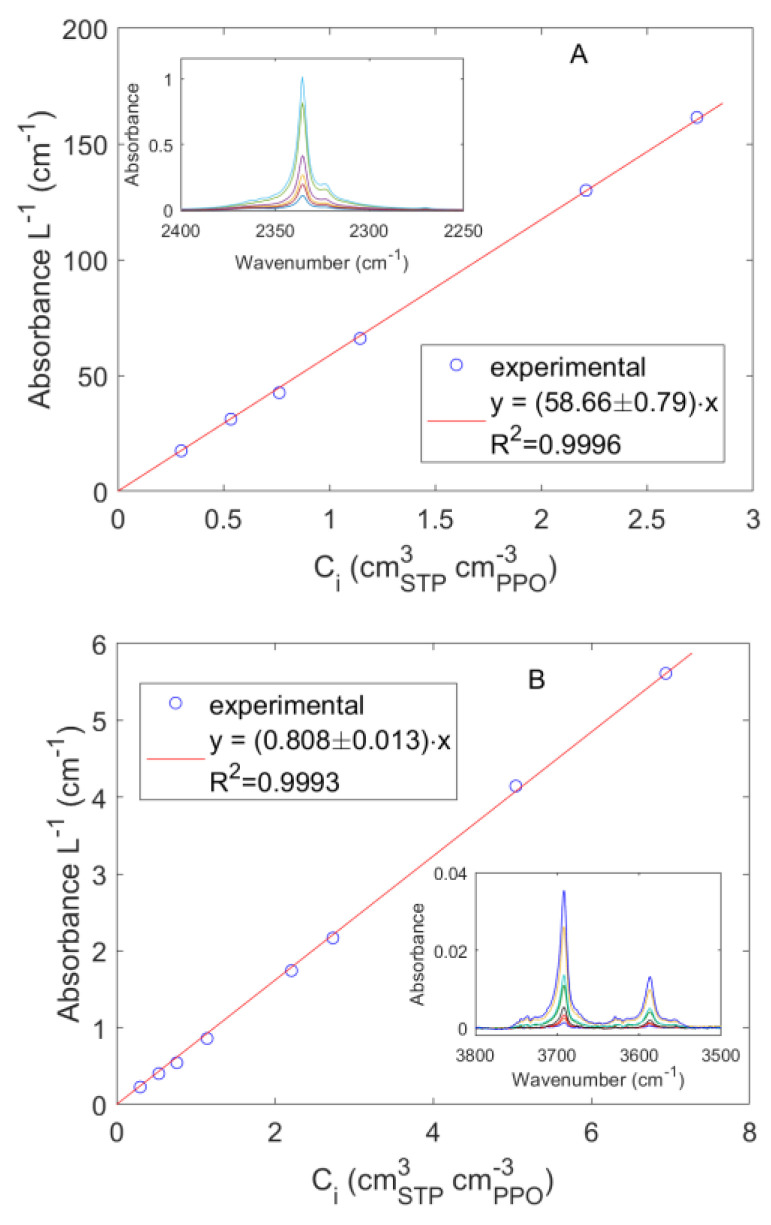
Correlation between the absorbance measured at 2 cm^−1^ frequency resolution and the concentration of carbon dioxide in PPO. (**A**): IR peak centered at 2336 cm^−1^; (**B**): IR peak centered at 3692 cm^−1^.

The calibration of the CO_2_ IR peaks at 2336 cm^−1^ and 3692 cm^−1^ was obtained from pure gas sorption tests by correlating the absorbance intensity with the concentration of CO_2_ within PPO (Figure 6). A linear trend was observed in both cases resulting, respectively, in an absorptivity of 58.66 and 0.808 cm^3^_PPO_ cm^−3^_STP_ cm^−1^. The CO_2_ peaks were symmetrical and well resolved as expected when no specific interactions were occurring with the polymer matrix.

**Figure 7:**
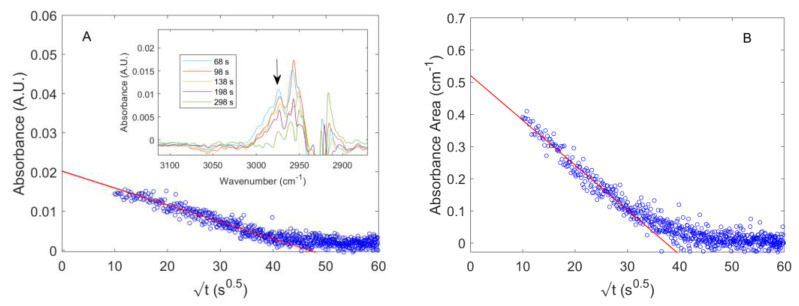
Integral desorption kinetics of CH_4_ from PPO. The initial pressure is equal to 753.5 Torr. (**A**) Absorbance at 2973 cm^−1^; (**B**) absorbance area in the range [2969,3005] cm^−1^.

In the case of methane absorbed in amorphous PPO, the subtraction of the gas phase background spectrum from the overall IR spectrum, at sorption equilibrium, did not provide any significant result in terms of signals amenable for any qualitative or quantitative analysis. However, it was possible to isolate the IR stretching vibration of CH_4_ absorbed in PPO by analyzing the desorption kinetics. This approach should be preferred when the signals to be isolated have low intensity and/or their absorptivity is still unknown. During an integral desorption step, a pressure jump down to 1 × 10^−3^Torr was produced in chamber 3 in approximately 100 s so that, after this time, the IR signals which identify the gas phase disappeared and those associated with the absorbed gas were unveiled in the same frequency region. In Figure 7A, the evolution with time of the absorbance of the signal at 2973 cm^−1^, during an integral desorption step starting from 753.5 Torr, is reported. In the inset, the sequence of spectra measured in the range [2850,3100] cm^−1^ was reproduced. After approximately 1 min from the valve opening, the gas phase signal was no more appreciable and the kinetics only represented the desorption of CH_4_ from PPO. In Figure 7B, the absorbance area evaluated in the frequency region [2969,3005] cm^−1^ is reported. The relative uncertainty was lower, and the sorption kinetics was better reproduced than in the case of absorbance. In both plots, the values were reported as a function of the square root of time (Fick’s plot). To the authors’ knowledge, this is the first time that methane is observed directly within a polymer phase. Li and Xi investigated the adsorption of methane on Cerium Oxide by means of FT-IR Spectroscopy at −100 °C [36]. Further, Yoshida et al. observed CH_4_ adsorbed on active carbon at −120 °C [37]. The identification of methane physiosorbed on porous materials or absorbed in dense materials is a rather difficult task at ambient temperature with FTIR Spectroscopy. We conducted further experiments to evaluate the absorptivity of the isolated signal for quantitative analyses. To gather a preliminary estimate of the absorptivity, we first extrapolated to t = 0 s, the plot of the values of the absorbance at 2973 cm^−1^ and of the absorbance area in the range [2969,3005] cm^−1^ (see Figure 7). These values were then divided by the concentration of methane in PPO (taken from Figure 3). The absorptivity of the signal at 2973 cm^−1^ was estimated to be 1.216 cm^3^_PPO_ cm^−3^_STP_ cm^−1^ while the absorptivity of the absorbance area in the range [2969,3005] cm^−1^ was estimated to be 31.65 cm^3^_PPO_ cm^−3^_STP_ cm^−2^.

### 4.2. Diffusion of Pure CO_2_ in PPO

The diffusivity of pure CO_2_ in PPO can be evaluated by fitting Equation (16) to the sorption kinetics measured with FTIR Spectroscopy. The temperature was not uniform throughout the closed volume, so the procedure outlined in the Section *Sorption of Pure CO_2_ and CH_4_ in PPO* for the solubility of each gas in PPO at equilibrium should be used to evaluate the sorption kinetics with the pressure decay method. Conversely, the sorption kinetics obtained from the time resolved IR spectrum of the gas species absorbed within the polymer was not affected by this constraint, the IR signal being measured at 35 °C. In addition, a further source of error in the case of the pressure decay method was the thickness variance among all the samples placed inside the measuring chamber as compared to the IR spectroscopy measurement that is instead performed on a single specimen, thus lowering, in this case, the uncertainty of the estimated value of diffusivity.

**Figure 8:**
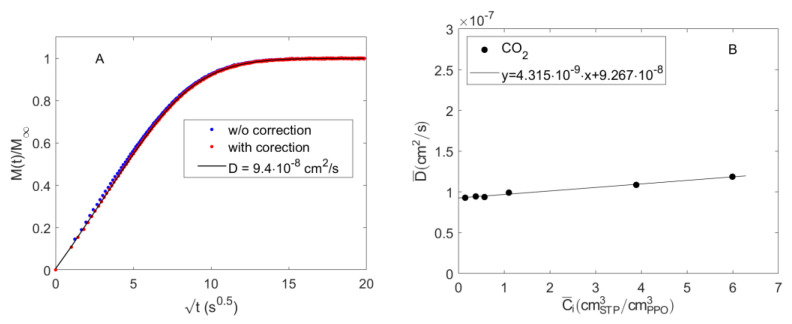
Sorption kinetics of CO_2_ in amorphous PPO. (**A**) IR integral sorption kinetics up to 98.56 Torr at 35 °C with and without the gas phase correction reported in Equation (18). The black continuous line is the fitting from Equation (16); (**B**) estimated values of effective diffusivity of carbon dioxide in amorphous PPO as a function of average CO_2_ concentration during each sorption step.

It is worth mentioning that the decrease of gas pressure during sorption introduces two distinct problems. First, Equation (16) represents the analytical solution to the diffusion problem in a plane sheet when the boundary conditions in terms of concentration at the surface of the sheet are kept constant. We have observed a pressure decrease of 4.3% and 0.8% relative to the pressure at sorption equilibrium during the tests at 24.49 and 756.2 Torr, respectively. Within this pressure range, the observed pressure variation was low enough to assume that Equation (16) was still valid. Second, to retrieve the IR signal of the species absorbed within the polymer during sorption, *difference spectroscopy* was used to remove the gas phase contribution (*A_gas_*) from the overall spectrum (*A*). To this aim, one should account for the fact that the IR signal of the gas phase was changing during time, so that the subtraction relation was rewritten as follows:

(17) $$A_{gas}\left(t\right)-k\left(t\right)\cdot A_{bkg}=0$$

where *A_bkg_* is the gas phase background spectrum measured at the same pressure and temperature reached at sorption equilibrium and corrected for the polymer specimen thickness; *k(t)* is a time dependent correction factor accounting for the fact that the thermodynamic conditions of the gas phase (pressure) at a generic time during sorption are different from those attained at sorption equilibrium (i.e., when *k(t→∞) = 1*). The time dependent correction is easily calculated from the classical Beer–Lambert law applied to Equation (17), as follows:

(18) $$k\left(t\right)=\frac{C^{g}\left(t\right)}{C_{\infty }^{g}}$$

where $C^{g}\left(t\right)$ and $C_{\infty }^{g}$ are, respectively, the gas phase concentration during time and at sorption equilibrium and are retrieved from the barometric measurement. In Figure 8A, the IR integral sorption kinetics of CO_2_ in PPO at 98.56 Torr, respectively, obtained applying the gas phase correction (according to Equation (18)) and without any correction (i.e., with *k(t) = 1*) are reported. The two results are very similar to each other. Equations (17) and (18) show the complementarity of barometry and FTIR Spectroscopy to measure the diffusivity of the species in the polymer from the IR signal of the species absorbed within the polymer. In fact, while FTIR Spectroscopy can be also used as a standalone technique to evaluate the sorption kinetics and the diffusivity of CO_2_ in PPO, the combination of the two techniques is essential to calibrate the IR signals. 

The value of the effective diffusivity of CO_2_ in PPO, $\bar{D}$, estimated from the fitting of the sorption kinetics data at 98.56 Torr, after applying the correction provided by Equation (17) (data in red in Figure 8A), was 9.4 × 10^−8^ cm^2^ s^−1^. The same procedure was adopted to estimate the values of $\bar{D}$ at all the investigated gas pressure conditions. In Figure 8B, these values are reported as a function of the average CO_2_ concentration during each sorption test. In the concentration range investigated, the diffusivity was almost constant, showing a slight linear increase. 

The effective diffusivity of CH_4_ in PPO was estimated from the desorption kinetics presented in Figure 7 and has been found to be equal to 6 × 10^−9^ cm^2^ s^−1^ at an average concentration of 2.11 cm^3^_STP_ cm^−3^_PPO_.

### 4.3. Modelling Sorption of Light Gases in PPO

The analysis of the IR bands of the polymer and the absorbed CO_2_ indicates that no specific interactions occur in the polymer–penetrant systems investigated. Based on that, sorption thermodynamics of both pure gases in amorphous PPO was interpreted using the *NETGP-NRHB* model without the inclusion of the terms accounting for the contribution of specific interactions. In the following, we refer to this simplified form of the model as *NETGP-NR* model. 

**Figure 9:**
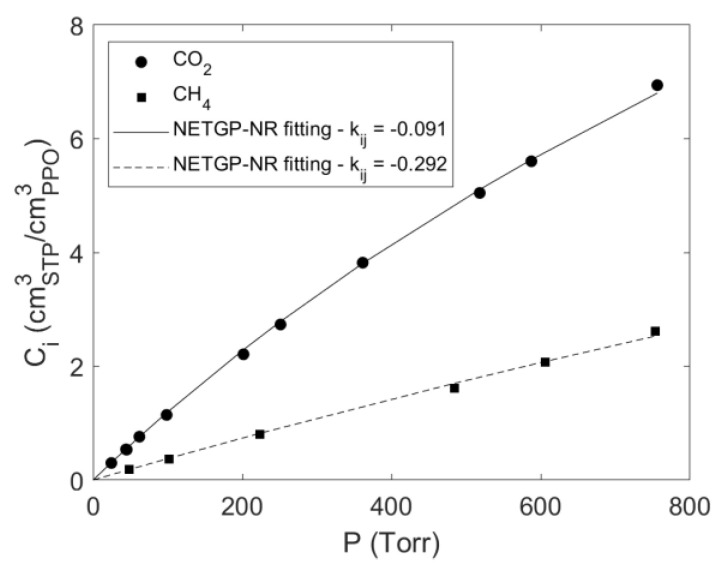
*NETGP-NR* model fitting of pure gas sorption in PPO (*k_sw_ = 0*).

The four *NR* composition-independent lattice fluid parameters of PPO, CO_2_ and CH_4_ have been taken from the literature and are reported in Table 1. Then, solubility data for CO_2_–PPO and CH_4_–PPO binary systems were fitted with the *NETGP-NR* model using the *k_ij_* binary interaction parameter as the fitting parameter. In Table 2, the estimated values of *k_ij_* are reported, and in Figure 9, the results of the fitting procedure are shown. 

In a previous publication, we modelled the binary solubility data at 35 °C of CO_2_ and CH_4_ in an amorphous PPO which, due to a different thermo-mechanical history, displayed a slightly different value of $\rho_{p,0}$ [16]. This set of data spans a higher range of pressure (up to 20 atm), so that a swelling effect, induced by the penetrant, was taken into account according to the phenomenological equation [19,24,25]:

(19) $$\rho_{p,0}^{sw}=\rho_{p,0}\cdot \left(1-k_{sw}P\right)$$

where $\rho_{p,0}^{sw}$ represents the actual polymer mass density to be used in the equations regarding the polymeric phase and $k_{sw}$ represents an additional binary adjustable parameter, which allows the expression of the elastic swelling contribution due to the absorbed penetrant in the glassy polymeric matrix.

**Table 1:** NR lattice fluid parameters.

	$\varepsilon_{i,h}^{*}$ (Jmol^−1^)	$\varepsilon_{i,s}^{*}$ (Jmol^−1^K^−1^)	$v_{i,sp,0}^{*}$ (cm^3^g^−1^)	$s_{i}$	Ref.
CO_2_	3468.4	−4.5855	0.79641	0.909	[38]
CH_4_	1956.2	−0.9181	2.12519	0.961	[38]
PPO	5320	3.440	0.862	0.748	[39]

In that case, extremely low values of *k_sw_* for PPO/CO_2_ and PPO/CH_4_ systems were determined (respectively, 1.96 × 10^−6^ Torr^−1^ and 4.48 × 10^−7^ Torr^−1^). On this basis, we assumed that the swelling induced by the two penetrants in the low range of pressures analyzed here was negligible, so that *k_sw_* = 0 can be safely fixed. This assumption was also confirmed by the outcome of the IR investigation indicating that no-swelling took place in the overall range of pressure investigated. The same assumption was adopted also in the following analysis of the solubility of CO_2_/CH_4_ mixtures within the PPO glassy matrix in view of the low values of pressure. The values of *k_ij_* obtained in the present investigation from fitting of the sorption isotherms by fixing *k_sw_* = 0 were in good agreement with those determined in Ref. [16], also reported in Table 2.

**Table 2:** *k_ij_* mean–field interaction parameter of pure CO_2_ and CH_4_ in PPO.

CO_2_	CH_4_	Ref.
−0.091	−0.292	This Work
−0.087	−0.278	[16]

### 4.4. Mixed Gas Sorption in PPO

**Figure 10:**
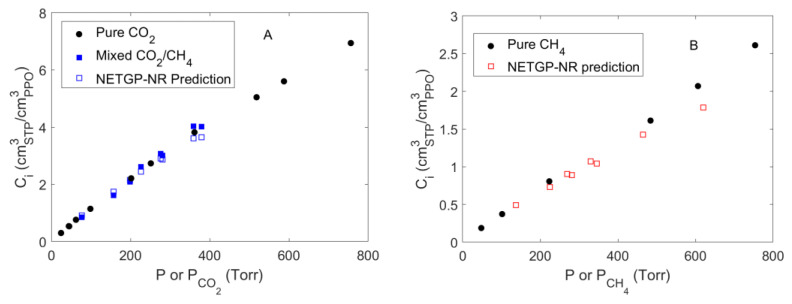
Pure and mixed gas sorption isotherms at 35 °C for CO_2_ (**A**) and CH_4_ (**B**) in amorphous PPO. Predictions are referred to mixture sorption and were obtained using *NETGP-NR* model.

Mixed gas sorption experiments were conducted at 35 °C, at values of total pressure up to 1000 Torr. Mixtures of only two molar compositions at several pressures were considered, i.e., CO_2_ mole fractions of ~0.50 and ~0.37 mol mol^−1^. In Figure 10, the solubility of each gas absorbed within the polymer is reported, comparing the results of pure gas and of mixture sorption experiments. In the case of pure gas sorption, the values of concentration of each gas at sorption equilibrium were reported as a function of the total pressure (indicated by the symbol *P*), while, in the case of mixture sorption, they were reported as a function of the partial pressure of each gas (indicated by the symbol *P_i_*). The partial pressure at sorption equilibrium was calculated based on the total pressure of the system and of the composition of the gas phase. The latter was measured spectroscopically by evaluating the concentration of CO_2_ and CH_4_ in the gas phase from the IR peaks located at 4991 and 4218 cm^−1^, respectively. At these frequencies, no IR bands associated with the polymer or with the absorbed species were present, so that the gas phase signals were well isolated and resolved and were used without any further treatment. The protocol proposed by Loianno et al. was followed to calibrate these signals [11,29].

We measured the concentration of carbon dioxide absorbed within the amorphous PPO (Figure 10A) using the IR signal of absorbed CO_2_ centered at 3692 cm^−1^ (calibrated using data in Figure 6B). Its absorptivity was assumed constant and independent of the composition of the polymer phase since no specific interactions were occurring with the polymer or the methane molecules absorbed in PPO. The numerical values are reported in Table S1 (see Supplementary Material). The solubility of CO_2_ in PPO was invariant up to a partial pressure equal to 379.0 Torr in the range of gas mixture composition investigated. Story and Koros conducted sorption experiments of CO_2_/CH_4_ gas mixtures in amorphous PPO by fixing the partial pressure of one gas and by changing the partial pressure of the other [7]. At a partial pressure of CO_2_ equal to 5.097 bar and CO_2_ mole fractions equal to 0.549 and 0.380 mol mol^−1^, they observed a reduction of the CO_2_ solubility with respect to pure gas sorption equal to 9.6% and 20%, respectively. Then, a significant deviation from the pure gas sorption isotherm was expected at CO_2_ partial pressure greater than 2 bar. 

We were not able to evaluate the solubility of CH_4_ in PPO since, at sorption equilibrium, no signal associated with methane absorbed in PPO can be resolved, in view of the interference of the CH_4_ signals associated with the gaseous phase surrounding the polymer sample.

Using the PPO/CO_2_ and PPO/CH_4_ binary parameters estimated from pure gas sorption tests and the value of *k_ij_* = 0.0406 for the system CO_2_/CH_4_ taken from the literature, we were able to predict, by means of the *NETGP-NR* model, the concentration of each gas absorbed at equilibrium in PPO in the case of mixture sorption (see empty square symbols in Figure 10A,B). The predicted solubility of CO_2_ in PPO from mixtures deviated at the most by 9.3% from the experimental values. This difference is within the uncertainty of both data sets and confirms the efficacy of the model to predict the behavior of such polymer–penetrant systems. In the case of CH_4_, we were not able to assess the reliability of model predictions since no data for methane sorption in the case of mixtures could be gathered from experiments. 

**Figure 11:**
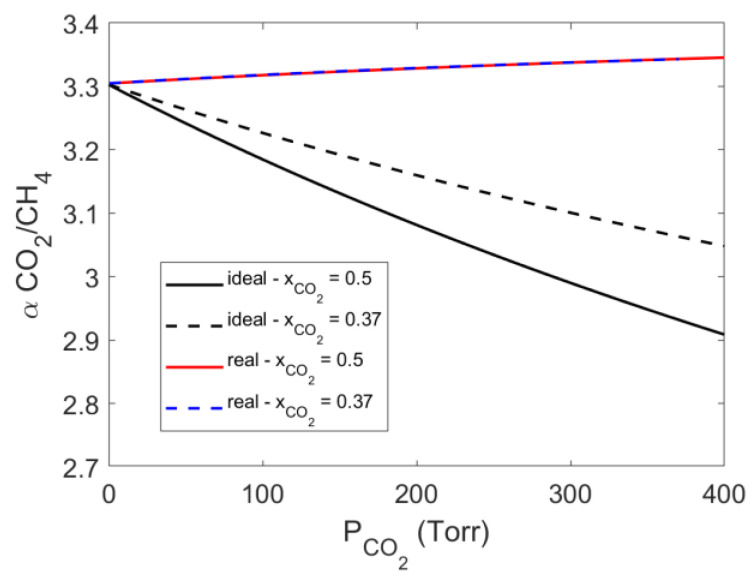
Comparison between ideal and real solubility selectivity as a function of carbon dioxide partial pressure and mole fraction. The solubility selectivity is calculated with the *NETGP-NR* model.

The use of *NETGP-NR* model in the case of mixture sorption allows to estimate the difference between the ‘ideal’ and the (predicted) ‘real’ solubility selectivity. In Figure 11, these are reported as a function of the partial pressure of CO_2_ in the case of the two molar compositions investigated here.

The ‘real’ solubility selectivity was found to be rather independent from the gas mixture composition within the narrow range of pressure investigated. Conversely, the ideal solubility selectivity was found to decrease as a function of CO_2_ partial pressure. In fact, by using the ideal selectivity, one implicitly assumes that no interference occurs between the two absorbed species, so that the two gases behave like if they were pure gases. In such a case, the form of the curves reported in Figure 11 reflects the relative shape of sorption isotherms for pure gases (CO_2_ sorption isotherms approach saturation at pressure values that are lower than in the case of CH_4_). Conversely, the ‘real’ selectivity accounts for the fact that the more condensable CO_2_ molecules displace the CH_4_ molecules from adsorption sites within the glassy polymer.

## 5. Conclusions

In this contribution, the sorption of carbon dioxide, methane and their mixtures in amorphous PPO was studied by performing experiments up to 1000 Torr at 35 °C. Barometry and FTIR Spectroscopy were combined to evaluate the solubility of each gas in the polymer. Relevant IR signals in the polymer phase were identified for each of the two penetrants and were also used to detect possible specific interactions

We identified several IR bands of carbon dioxide absorbed in PPO, which were conveniently calibrated over the penetrant concentration by comparison with pure gas barometric sorption tests. These signals were useful to evaluate the concentration of CO_2_ in PPO in the case of mixed gas sorption tests. Conversely, for absorbed CH_4_, although we identified an IR band suitable for qualitative and quantitative analysis, this could be used only to investigate pure gas desorption kinetics and not in the case of pure gas and mixture sorption, in view of an interference with signals related to methane in the gas phase. FTIR spectroscopy also allowed us to evaluate the diffusivity of CO_2_ in amorphous PPO. The main result we obtained in the case of CO_2_/CH_4_ mixtures is the invariance of the carbon dioxide solubility up to a CO_2_ partial pressure of 450 Torr, at 35 °C and CO_2_ mole fractions equal to ~0.37 and ~0.5.

Sorption isotherms, in PPO, of CO_2_, CH_4_ and their mixtures were successfully interpreted using the *NETGP-NR* thermodynamic model. The model requires one binary parameter for each couple of components and only accounts for “mean field” energy interaction. This assumption is supported by the indication of the IR analysis that excludes the occurrence of specific interactions between the polymer chains and the absorbed molecules. The binary interaction parameters of the model have been first determined by fitting pure gases sorption isotherms. Then, using these values, we tested the capability of the model to predict the solubility of the corresponding binary gas mixtures in PPO. This assessment was possible only in the case of carbon dioxide. The prediction provided for CO_2_ solubility slightly deviates from the experimental data set (<9.3%) and indicates that, in contrast with the increase of the ideal sorption selectivity as the partial pressure of carbon dioxide in the mixture increases, the ‘real’ (predicted) solubility selectivity is rather constant with CO_2_ partial pressure and mole fraction.

## Data Availability

New data generated in this investigation are reported in Table S1 of the Supporting Information.

## Supplementary Materials

The following supporting information can be downloaded at: https://www.mdpi.com/article/10.3390/polym15051144/s1, Table S1: Mixed gas sorption experiments: solubility of CO_2_ (component #1) in amorphous PPO.

## List of Symbols

$A_{gas}\left(t\right)$	gas phase contribution to the overall absorbance spectrum at time *t*
$A_{bkg}$	gas phase contribution to the overall absorbance spectrum at sorption equilibrium state
*C*	molar concentration of the pure penetrant
$C^{d}$	molar concentration of the pure penetrant in the polymer phase at the downstream side in a permeation experiment
$C^{up}$	molar concentration of the pure penetrant in the polymer phase at the upstream side in a permeation experiment
$C_{i}$	concentration of component *i* within the polymer phase in a sorption experiment
${\bar{C}}_{i}$	average concentration of component *i* within the polymer phase in a sorption experiment
$C_{i}^{0}$	initial uniform concentration of component *i* within the polymer phase in a sorption experiment
$C_{i}^{\infty }$	equilibrium concentration of component *i* within the polymer phase in a sorption experiment
$C^{g}\left(t\right)$	molar gas phase concentration at time *t*
$C_{\infty }^{g}$	molar gas phase concentration at sorption equilibrium state
*D*	mutual diffusion coefficient
*D_i_*	mutual diffusion coefficient of the component *i*
$\bar{D}$	effective diffusivity coefficient
$k\left(t\right)$	defined in Equation (17) and calculated from Equation (18)
$k_{ij}$	mean field lattice fluid binary interactional parameter between the species *i* and *j*
$k_{sw}$	the swelling factor associated with the polymer-penetrant couple
$l_{i}$	defined as $\frac{z}{2}\left(r_{i}-q_{i}\right)-\left(r_{i}-1\right)$
*L*	membrane thickness in a sorption experiment
m	number of components
$M\left(t\right)$	mass of absorbed penetrant at time *t* in a sorption experiment
$M_{\infty }$	equilibrium mass of absorbed penetrant in a sorption experiment
$P_{i}^{up}$	partial pressure of the component *i* at the upstream side in a permeation test
*P*	pressure
$\tilde{P}$	scaled pressure of the pure component or of the mixture
$\bar{Perm}$	mean steady-state permeability
$Perm_{i}$	steady-state permeability of component *i*
*q*	average number of lattice contacts per molecule in the mixture and it is equal to $\sum_{i=0}^{m}x_{i}q_{i}$
*q_i_*	number of external contacts made by one molecule of species *i*
*R*	universal gas constant
*r*	average number of sites occupied by one molecule in the mixture and it is equal to $\sum_{i=0}^{m}x_{i}r_{i}$
*r_i_*	number of sites occupied by one molecule of species *i*
*S*	apparent solubility coefficient
$S_{i}^{mix}$	solubility coefficient of component *i* in the polymer–penetrant mixture
*t*	time
*T*	temperature
*T_g_*	glass-to-rubber transition temperature
$\tilde{T}$	scaled temperature of the pure component or of the mixture
${\tilde{T}}_{i}$	scaled temperature of the pure component *i* in the mixture
$v_{i,sp,0}^{*}$	the temperature-independent contribution to the close packed specific volume of the pure component *i*
$v_{i,sp}^{*}$	close packed specific volume of the pure component *i*
$\tilde{v}$	scaled lattice fluid volume of the pure components or of the mixture and it is equal to $1/\tilde{\rho }$
$s_{i}$	the ratio of molar surface to molar volume of component *i*
*x_i_*	molar fraction of species *i*
$x_{i}^{NE}$	molar fraction of species *i* at Pseudo-Equilibrium of phase condition
*z*	lattice coordination number
Greek letters	
$\alpha^{mix}$	solubility selectivity defined by Equation (15)
*α^id^*	ideal selectivity
$\alpha_{S}^{id}$	solubility contribution to the ideal selectivity
$\alpha_{D}^{id}$	diffusivity contribution to the ideal selectivity
$\Gamma_{ij}$	non-random factor
$\Delta \varepsilon_{ij}$	exchanged interaction energy determined by breaking one i-ì and one j-j interaction for the formation of two i–j interactions in the lattice
$\delta_{i}$	flexibility factor of species *i*
$\varepsilon^{*}$	mean field interaction energy within the mixture
$\varepsilon_{i}^{*}$	mean field interaction energy per molar segment
$\varepsilon_{i,h}^{*}$	mean interaction energy per molar segment, enthalpic contribution
$\varepsilon_{i,s}^{*}$	mean interaction energy per molar segment, entropic contribution
$\Theta_{i}$	surface fraction of component *i*, $\theta_{i}=\frac{x_{i}q_{i}}{q}$
$\mu_{i}$	molar chemical potential of the species *i*
$\mu_{i,ext}^{EQ}$	equilibrium molar chemical potential of the species *i*
$\mu_{i,pol}^{NE}$	non-equilibrium molar chemical potential of the species *i* within the glassy polymer–penetrant phase
ρ	non-equilibrium polymer mass density within the mixture
$\rho^{*}$	closed-packed density of the polymer–penetrant mixture
$\rho_{p,0}^{sw}$	polymer mass density induced by elastic instantaneous swelling
$\rho_{p,0}$	the value of the unpenetrated polymer mass density right before the start of the sorption process
$\tilde{\rho }$	scaled lattice fluid density of a pure component or of the mixture ($=\rho /\rho^{*})$
$\Phi_{i}$	“close-packed” volumetric fraction of component *i*
$\omega_{p}$	polymer mass fraction
