# Supplementary material for: Sorption of CO2, CH4 and Their Mixtures in Amorphous Poly(2,6-dimethyl-1,4-phenylene)oxide (PPO)
Source: Polymers (Basel). 2023 Feb 24;15(5):1144. doi: 10.3390/polym15051144 (PMC10007344; doi:10.3390/polym15051144)
Supplement: Supplementary file 1 [file polymers-15-01144-s001.zip › polymers-2193451-supplementary1.pdf]

Supplementary Material

Sorption of CO_2, CH_4 and their mixtures in Amorphous Poly(2,6-dimethyl-1,4-phenylene)oxide (PPO)

Valerio Loianno, Antonio Baldanza, Giuseppe Scherillo, Pellegrino Musto and Giuseppe Mensitieri

Table S1. Mixed gas sorption experiments: solubility of CO_2 (component #1) in amorphous PPO.

T (°C)	P (Torr)	x_1 (mol mol^{-1})	C_1 (cm$^3_{STP}$ cm$^{-3}_{PPO}$)
35.00±0.02	214.3±1.1	0.360	0.849
35.00±0.02	426.8±2.1	0.368	1.611
35.00±0.02	528.8±2.6	0.376	2.087
35.00±0.02	745.8±3.7	0.377	3.014
35.00±0.02	999.1±5.0	0.379	4.020
35.00±0.02	450.2±2.2	0.502	2.615
35.00±0.02	557.4±2.8	0.495	3.078
35.00±0.02	705.2±3.5	0.510	4.036
